# Profiling of small RNAs derived from tomato brown rugose fruit virus in infected *Solanum lycopersicum* plants by deep sequencing

**DOI:** 10.3389/fmicb.2024.1504861

**Published:** 2025-01-30

**Authors:** Mangle Chen, Donghai Wang, Jiali Yang, Yuhao Cao, Xuemei Song, Yuwen Lu, Hongying Zheng, Jiejun Peng, Guanwei Wu, Jian Wu, Junmin Li, Jianping Chen, Fei Yan, Shaofei Rao

**Affiliations:** State Key Laboratory for Quality and Safety of Agro-Products, Key Laboratory of Biotechnology in Plant Protection of MARA, Zhejiang Key Laboratory of Green Plant Protection, Institute of Plant Virology, Ningbo University, Ningbo, China

**Keywords:** tomato brown rugose fruit virus, RNA silencing, virus-derived small interfering RNA, high-throughput sequencing, tomato

## Abstract

Tomato brown rugose fruit virus (ToBRFV) is an emerging, rapidly spreading virus belonging to the genus *Tobamovirus* which seriously decreases tomato yields. RNA silencing is an evolutionarily conserved antiviral mechanism. In virus-infected plants, virus-derived small interfering RNAs (vsiRNAs) are one of the key components involved in the RNA silencing-based antiviral activity in plants. The main function of vsiRNAs is to target and degrade viral RNA. Studies have found that some vsiRNAs can also target host transcripts, further regulating host responses and symptoms and promoting viral survival and spread. In this study, the vsiRNA profiles of ToBRFV-infected tomato plants were obtained by deep sequencing. VsiRNAs were mainly 21 and 22 nucleotides in length and had a U-bias at the 5′ end. The single-nucleotide resolution profile shows that vsiRNAs exhibit a heterogeneous continuous distribution in the ToBRFV genomic RNA, with hotspot regions on the antisense strand located at the 5′ end of the RdRP and in the coding regions of MP and CP. The presence of vsiRNAs was confirmed in tomato plants infected with ToBRFV through RT-PCR, and GO and KEGG enrichment analyses were performed on the predicted vsiRNA target genes (with an expectation value less than or equal to 2.5). Seven potential target genes were selected for qRT-PCR analysis, confirming that their transcript accumulation significantly decreased in the leaves of tomato plants infected with ToBRFV. These genes may play an important role in the process of viral infection in tomatoes. Our results suggest a role for vsiRNAs in the ToBRFV–tomato interaction.

## 1 Introduction

RNA silencing, or RNA interference, is a gene-silencing mechanism that is widely present in eukaryotes (Shabalina and Koonin, 2008; Weiberg and Jin, 2015). It plays a crucial role in developmental regulation, stress responses, and host defense against transposons and viruses (Castel and Martienssen, 2013; Martínez de Alba et al., 2013; Pumplin and Voinnet, 2013). RNA silencing in plants is induced by double-stranded or highly structured single-stranded RNAs that are processed by Dicer-like ribonucleases (DCLs) to produce small RNA with lengths of 21–30 nucleotides (nt) (Zhu and Guo, 2012). During plant RNA virus infection, double-stranded viral replication intermediates (RI) or secondary structured single-stranded RNA genomic regions can initiate DCL-mediated biogenesis of primary virus-derived siRNA (vsiRNA) (Ahlquist, 2002; Molnár et al., 2005; Donaire et al., 2009; Szittya et al., 2010). The vsiRNA duplex subsequently guides Argonaute (AGO) proteins to form the RNA-induced silencing complex (RISC), which degrades the target complementary viral RNA through a slicing mechanism (Baumberger and Baulcombe, 2005; Mi et al., 2008). In virus-infected plants, the effects of RNA silencing can be amplified when RNA-dependent RNA polymerases (RDRs) produce secondary vsiRNAs derived from aberrant viral dsRNA (Wang et al., 2009). Many DCLs, AGOs, and RDRs are involved in plant antiviral defenses based on RNA silencing. In *Arabidopsis thaliana*, DCL2 and DCL4 play a key role in the production of positive RNA virus-derived siRNAs. In plants infected with positive RNA viruses, 21-nt vsiRNAs are mainly processed by DCL4 (Bouché et al., 2006; Fusaro et al., 2006; Garcia-Ruiz et al., 2010). However, in the absence of DCL4, the size of vsiRNAs appears to shift toward the 22-nt species that depend on DCL2 (Deleris et al., 2006). DCL3 produces 24-nt vsiRNAs only in *dcl2/dcl4* double mutants and therefore appears to play a secondary role in this process (Bouché et al., 2006; Deleris et al., 2006; Fusaro et al., 2006; Garcia-Ruiz et al., 2010). DCL1 is currently believed to be only indirectly involved in the production of vsiRNAs (Zhu and Guo, 2012). By contrast, when DNA viruses infect plants, all four DCLs are involved in the production of vsiRNAs, among which DCL3 is relatively the most active antiviral Dicer, while DCL1 seems to assist the other DCLs (Blevins et al., 2006). Plant AGOs related to vsiRNAs include AGO1, AGO2, AGO4, AGO5, AGO7, AGO10, and AGO18, but AGO1 and AGO2 are the two main plant antiviral AGOs targeting RNA viruses (Wang et al., 2011; Carbonell and Carrington, 2015; Carbonell, 2017). *Arabidopsis* has six RDR proteins, and studies have shown that RDR1 and RDR6 may be involved in the biogenesis of secondary vsiRNAs (Baulcombe, 2004).

Current research shows that some vsiRNAs can also induce the degradation of host transcripts at the post-transcriptional level through base-pairing mechanisms, thereby inducing viral symptoms and facilitating viral invasion (Shimura et al., 2011; Smith et al., 2011; Navarro et al., 2012; Adkar-Purushothama et al., 2015; Yang et al., 2020). The vsiRNA of cucumber mosaic virus satellite Y cleaves the *CHL1* mRNA in *Nicotiana benthamiana*, which is involved in chlorophyll biosynthesis, leading to yellowing symptoms (Shimura et al., 2011; Smith et al., 2011). Navarro et al. found that two siRNAs of peach latent mosaic viroid (PLMVd; *Pelamoviroid latenspruni*) specifically targeted host *chloroplast heat shock protein 90* (*cHSP90*) transcripts, causing the peach leaves infected with PLMVd to show symptoms of albinism (Navarro et al., 2012). sRNAs from the toxic regulatory region of potato spindle tuber viroid (PSTVd; *Pospiviroid fusituberis*) target several callose synthase genes (*CalS11-like* and *CalS12-like*) in tomato plants, affecting callose formation during pathogen infection (Adkar-Purushothama et al., 2015). Chinese wheat mosaic virus (CWMV; *Furovirus chinense*) vsiRNA-20 regulates *TaVP* expression to prevent cell death and maintains a weakly alkaline environment in the cytoplasm to enhance CWMV infection in wheat (Yang et al., 2020).

Tomatoes are important field and greenhouse vegetable crops worldwide (Quinet et al., 2019). Among various damaging pathogens, viruses are one of the main hindrances to tomato production. Viral infections can lead to a decrease in tomato yield, resulting in significant economic losses (Hanssen et al., 2010; Hanssen and Lapidot, 2012; Jones and Naidu, 2019). Tomato brown rugose fruit virus (ToBRFV; *Tobamovirus fructirugosum*) is a newly identified virus isolated from tomatoes. The ToBRFV genome is approximately 6.4 kb in length and encodes four open reading frames (ORFs). ORF1 and ORF2 are directly translated from the genomic RNA and encode the 126 kDa and 183 kDa replication proteins, respectively. ORF3 and ORF4 are expressed from subgenomic RNAs and encode the movement protein (MP) and the coat protein (CP), respectively (Salem et al., 2016; Luria et al., 2017). Tomato plants infected with ToBRFV initially have mottled, yellow, and deformed leaves, while later, necrotic spots or brown streaks may appear on the fruit (Zhang et al., 2022; Salem et al., 2023). Studying the interaction between ToBRFV and tomato plants is crucial for developing effective antiviral strategies and ensuring the sustainability of tomato production.

In recent years, the vsiRNAs profiles of various RNA and DNA viruses in their host plants have been analyzed using deep sequencing technology (Yan et al., 2010; Mitter et al., 2013; Xia et al., 2014; Li et al., 2016; Li et al., 2017; Qiu et al., 2017; Xu and Zhou, 2017; Zhao et al., 2018; Wu et al., 2019; Lan and Lu, 2020; Jiao et al., 2022), but the siRNAs derived from ToBRFV have yet to be analyzed. In this study, high-throughput sequencing technology was used to identify and characterize vsiRNAs profiles in ToBRFV-infected tomato plants. The characteristics of vsiRNAs were analyzed, and target genes of some vsiRNAs were predicted. These data on the origin, distribution, and abundance of vsiRNAs obtained by high-throughput sequencing not only provide insights into the role of vsiRNAs in ToBRFV–tomato interaction but will also help design siRNA-mediated strategies against ToBRFV.

## 2 Materials and methods

### 2.1 Plant cultivation and virus inoculation

Tomato plants (variety: Moneymaker) were cultured in a greenhouse at 25°C with a 16-h light/8-h darkness photoperiod. Fourteen days after transplantation, healthy tomato plants were inoculated with ToBRFV by rubbing infected sap on the leaves, while control plants were treated with phosphate-buffered saline. The ToBRFV strain was isolated from Yuanmou County in Yunnan Province, China. Nineteen days post-inoculation, the plants infected with ToBRFV exhibited symptoms such as leaf narrowing, curling, blistering, and stunted growth, while the control group developed normally. Prior to sampling, RT-PCR confirmed the presence of ToBRFV in the newly emerged leaves of the inoculated group, whereas the negative control group showed no signs of ToBRFV infection. Three biological replicate samples (newly emerged top leaves from separate plants) were collected from each of the inoculated and control groups 19 days post-infection. These were sent to Beijing Novogene Bioinformatics Technology Co., Ltd. for small RNA sequencing.

### 2.2 Construction and sequencing of small RNA libraries

Samples of tomato leaves from the mock and ToBRFV-infected plants were used to extract total RNA using TRIzol reagent. The RNA was then treated with DNase I and analyzed for concentration, integrity, and overall quantity using the Agilent Bioanalyzer 2100 system. After confirming the quality of the samples, the NEB Next^®^ Multiplex Small RNA Library Prep Set for Illumina^®^ (NEB E7300L) was used to construct six libraries. In brief, the unique structures at the 3′ and 5′ ends of small RNAs were utilized (with a complete phosphate group at the 5′ end and a hydroxyl group at the 3′ end). Total RNA was used as the starting material, and adapters were directly added to both ends of the small RNAs. Reverse transcription primers were then hybridized to synthesize the first-strand cDNA. Following this, PCR amplification was performed, and the target DNA fragments were separated by PAGE gel electrophoresis. The desired DNA fragments were excised from the gel and recovered, resulting in the cDNA library. Once the quality of the library was confirmed, different libraries were multiplexed according to the required effective concentration and target sequencing depth before proceeding to sequencing.

### 2.3 Characteristic analysis of vsiRNAs

A script was written in Python 3 for data processing, including extracting sRNAs of 18–30 nt, counting the number of sRNAs of different lengths, and analyzing the types and quantities of bases at the 5′ ends of the sRNAs. Subsequently, an R script was used for visualization. Bowtie software (version 1.3.0) was employed to build an index of the viral genome, and then, Bowtie was used to align the sRNAs to the viral genome (allowing for one mismatch). The ToBRFV genomic sequence used for mapping was determined by our laboratory (isolated from diseased pepper samples in Yunnan Province, China), and its sequence has 99.6% identity to the published sequence of ToBRFV-SD (accession number: MT018320.1). From the SAM files generated during the Bowtie alignment, the number of aligned vsiRNAs at each base of the viral genome was counted, and an R script was written to create a distribution map of the vsiRNAs across the genome.

### 2.4 Prediction and enrichment analysis of vsiRNAs target genes

vsiRNAs with read counts greater than 2000 in ToBRFV-infected samples were selected for target gene prediction using the online target gene prediction tool psRNATarget^1^ (Tomato Genome Link^2^), with all parameters set to default. The predicted target genes were then subjected to GO and KEGG enrichment analysis using ClusterProfiler (4.6.0), followed by visualization through an R script.

### 2.5 RT-PCR detection of vsiRNAs

Total RNA was extracted from mock or ToBRFV-infected tomato leaves using the TRIzol method. The extracted RNA was then reverse-transcribed into cDNA using the miRcute Enhanced miRNA First-Strand cDNA Kit (TianGen). Using the obtained cDNA as a template, PCR amplification was performed with the miRcute Enhanced miRNA Fluorescent Quantitative Detection Kit (TianGen). The PCR conditions were as follows: denaturation at 95°C for 3 min, followed by 30 cycles of 95°C for 30 s, 55°C for 30 s, and 72°C for 20 s, with a final extension at 72°C for 7 min. Tomato U6 snRNA was used as an endogenous control. The PCR products were separated by 1% agarose gel electrophoresis and visualized under a gel imaging system (primer sequences are provided in Supplementary Table S1).

### 2.6 Real-time quantitative RT-PCR

Approximately 1 μg of total RNA was reverse-transcribed into cDNA using the All-in-One First-Strand cDNA Synthesis SuperMix for qPCR (One-Step gDNA Removal, Transgene) according to the manufacturer’s instructions. The resulting cDNA was then subjected to quantitative PCR using PerfectStart™ Green qPCR SuperMix (Transgene) on a Roche LightCycler^®^480 real-time PCR instrument (Roche Diagnostics). The intron-spanning quantitative PCR primers were designed based on the tomato genomic information provided by the Solanaceae Genomics Network^3^ and were confirmed for usability through RT-PCR (primer sequences are provided in Supplementary Table S1). *SlActin* was used as the internal reference gene, and the relative gene expression levels were calculated using the 2^−ΔΔCT^ method (Livak and Schmittgen, 2001).

## 3 Results

### 3.1 Deep sequencing of small RNAs

To identify the vsiRNAs involved in the interaction between ToBRFV and tomato, we constructed six small RNA libraries from phosphate-buffered saline-treated (mock) and ToBRFV-inoculated tomato leaves (three libraries per treatment, Table 1; Supplementary Figure S1). High-throughput sequencing of small RNAs generated 39,344,864 and 35,201,077 raw reads from the mock and ToBRFV libraries, respectively (Table 1). After removing low-quality sequences, unknown bases, polyA tails, and adapter sequences, we obtained 38,575,568 and 34,757,619 clean reads of lengths ranging from 18 to 30 nt from the mock and ToBRFV libraries, respectively. The number of unique reads was 4,086,743 and 3,359,072, respectively (Table 1). These reads were then aligned to the sense and antisense strands of the ToBRFV genome with a mismatch value set to 1 (the encapsidated genome of ToBRFV is the sense strand, but vsiRNAs can be sense or antisense with respect to the viral genome), and the resulting sequences were considered to be vsiRNAs. Ultimately, a total of 13,411,201 vsiRNAs were identified in the ToBRFV-inoculated sample library, with 357,316 unique read types, accounting for 38.6% of the total siRNA reads (Table 1). In contrast, only 2,631 sequences matched to the ToBRFV genome in the mock-inoculated library, representing approximately 0.007% of the 18–30 nt sequences (Table 1). This indicates that ToBRFV infection leads to the accumulation of vsiRNAs.

**Table 1:** Summary of deep sequencing results of small RNA libraries from tomato leaves inoculated with ToBRFV and mock treatment.

Category	ToBRFV-inoculated tomato plants			Mock-inoculated tomato plants		
	To-1	To-2	To-3	Mock-1	Mock-2	Mock-3
Raw reads	11,254,845	13,494,489	10,451,743	11,875,726	16,630,957	10,838,181
Clean reads	11,114,298	13,357,230	10,286,091	11,723,643	16,290,876	10,561,049
Unique reads	975,287	1,397,852	985,933	1,450,305	1,638,708	997,730
vsiRNA (Total reads)	4,072,724	5,207,731	4,130,746	487	808	1,336
vsiRNA (Unique reads)	110,827	137,097	109,392	378	597	849

**Figure 1:**
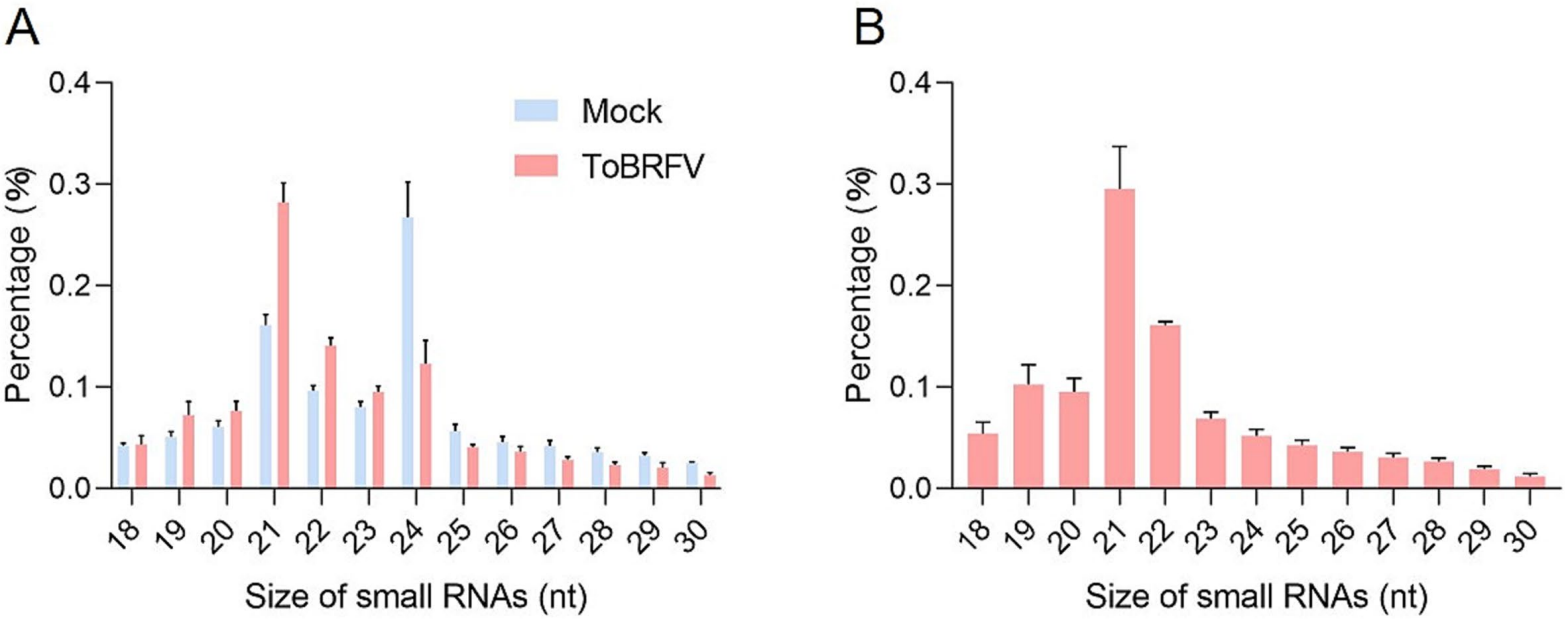
Infection by ToBRFV affects the overall size distribution of small RNAs in tomato leaves. **(A)** Size distribution of total small RNAs in mock-inoculated and ToBRFV-inoculated tomato plants. **(B)** Distribution of vsiRNAs for each size category in ToBRFV-infected tomato plants. Error bars indicate ± SD calculated from three biological replicates.

The comparative analysis of the small RNA profiles between the mock and ToBRFV libraries shows that ToBRFV infection significantly affected the overall size distribution of small RNAs in tomato leaves (Figure 1A; Supplementary Table S2). 21-nt and 24-nt small RNAs dominated in the mock library, whereas in the ToBRFV-inoculated library, there were more 21-nt and 22-nt sRNAs and significantly less 24-nt sRNAs (Figure 1A). In the infected plants, 21-nt and 22-nt vsiRNAs were the most abundant, accounting for 29.6 and 16.1% of the total vsiRNAs, respectively (Figure 1B). This suggests that the homologs of DCL4 and DCL2 in tomatoes may play a key role in the biogenesis of vsiRNAs and are important in the response to ToBRFV infection.

### 3.2 Characteristics of vsiRNAs

**Figure 2:**
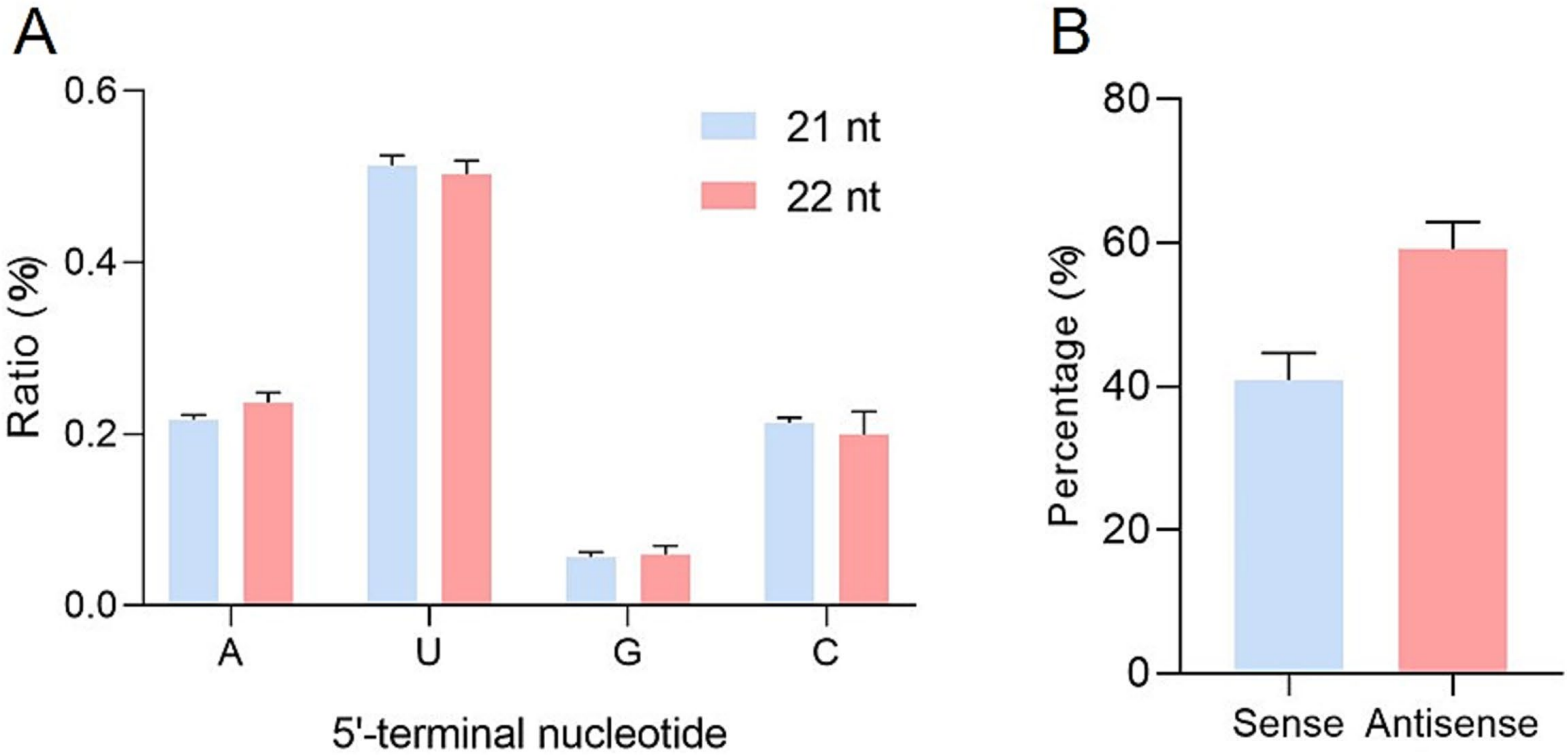
Relative frequencies of nucleotides at the 5′ end of vsiRNAs and the ratio of sense and antisense vsiRNAs derived from ToBRFV. **(A)** Relative frequencies of different nucleotides at the 5′ end of 21-nt and 22-nt vsiRNAs derived from ToBRFV. **(B)** Ratio of sense and antisense vsiRNAs derived from ToBRFV. Error bars indicate ± SD calculated from three biological replicates.

Small RNAs can bind to Argonaute (AGO) proteins to form effector complexes, collectively known as RNA-induced silencing complexes (RISCs). In *Arabidopsis*, the selective loading of small RNAs onto specific AGOs is influenced by their 5′ terminal nucleotides (Mi et al., 2008). AGO1 preferentially recruits small RNAs with a 5′ terminal uridine, while AGO2 and AGO4 have a high binding affinity for small RNAs with a 5′ terminal adenine, and AGO5 primarily binds to small RNAs that start with cytosine (Mi et al., 2008). To determine the potential interactions between ToBRFV-derived siRNAs and different AGO complexes, the relative abundance of vsiRNAs was analyzed based on their 5′ terminal nucleotides. Among the 21-nt and 22-nt vsiRNAs, U was the most frequent 5′ terminal nucleotide (about 50%), with approximately 20% of each being A and C and only approximately 5% G (Figure 2A). These findings suggest that 21-nt and 22-nt vsiRNAs may be potentially loaded into different AGO-containing complexes, with most vsiRNAs preferentially loading into AGO1/AGO2/AGO4/AGO5 homologs.

**Figure 3:**
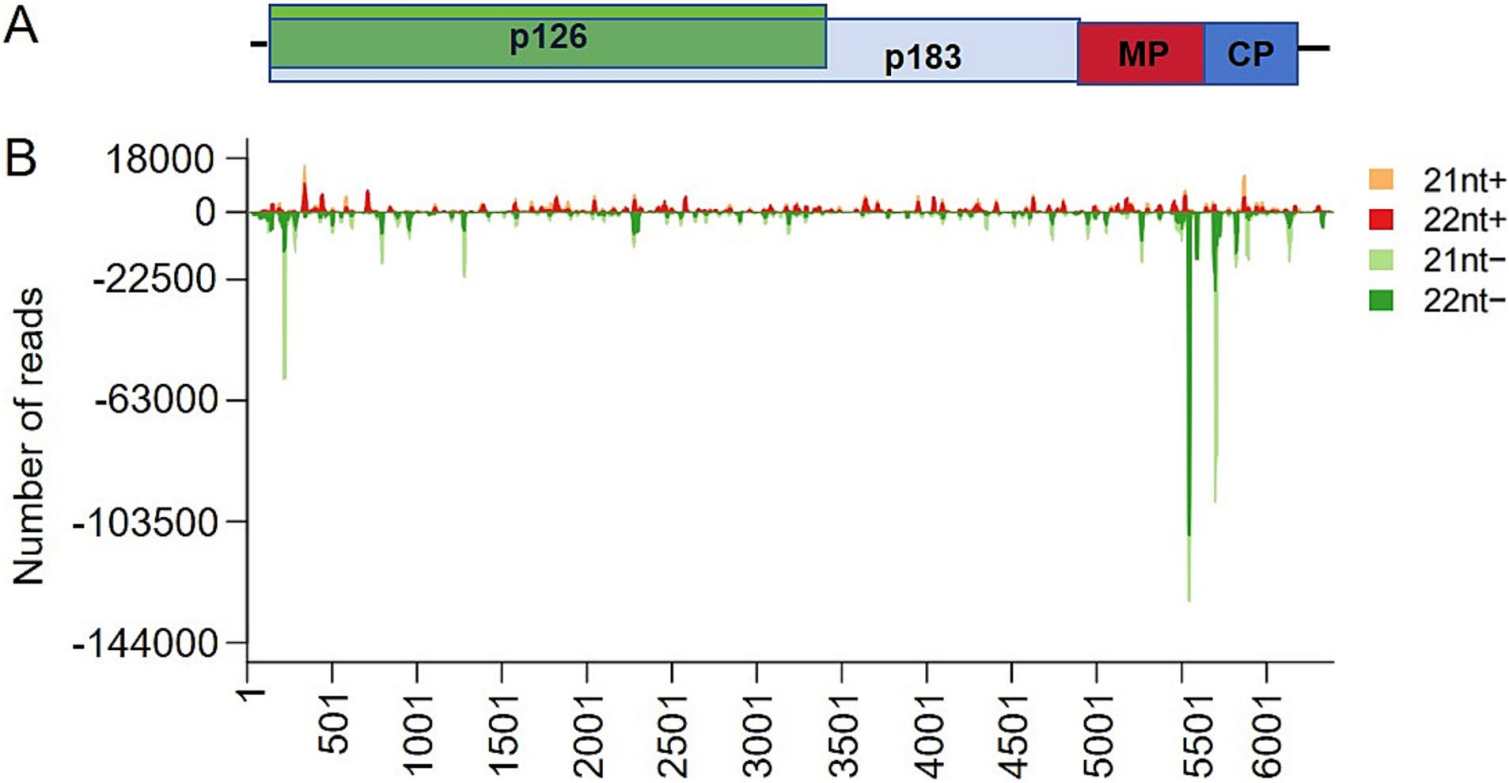
Distribution of ToBRFV-derived siRNAs along the viral genome. **(A)** Schematic diagram of the ToBRFV genome. **(B)** Distribution of 21-nt and 22-nt vsiRNAs in the ToBRFV genome at single-nucleotide resolution. This figure shows the number of vsiRNA reads at each nucleotide position in the 6,391-nucleotide ToBRFV genome. The ToBRFV genome is arranged along the x-axis and drawn to scale. vsiRNAs derived from the sense and antisense strands of ToBRFV are displayed above and below the horizontal line, respectively, with the Y-axis representing the number of reads. Three biological replicates obtained similar results, and the figure shows a representative result.

Further analysis showed that vsiRNAs derived from the antisense strand (59.13%) were more prevalent than those derived from the sense strand (40.87%) (Figure 2B). When matched to the ToBRFV genome, vsiRNAs from both polarities were distributed throughout the entire ToBRFV genome (Figures 3A,B; Supplementary Table S3) but with hotspot peaks of 21-nt and 22-nt vsiRNAs in the same genomic regions. To better understand the hotspots of vsiRNA distribution, we summarized and counted the number of reads in the single-nucleotide resolution maps for 21-nt and 22-nt vsiRNAs, defining regions with more than 30,000 vsiRNA reads as hotspots. There were no hotspots on the sense strand, while the antisense strand had three and one hotspot(s) for 21-nt and 22-nt vsiRNAs, respectively (Supplementary Table S4). These four hotspots are located in the regions corresponding to the p126 N-terminus, MP, and CP genes, with the position 837–859 on the antisense strand (the corresponding genomic sense strand position is 5533–5555) being a common hotspot for both 21-nt and 22-nt vsiRNAs (Figure 3B).

**Figure 4:**
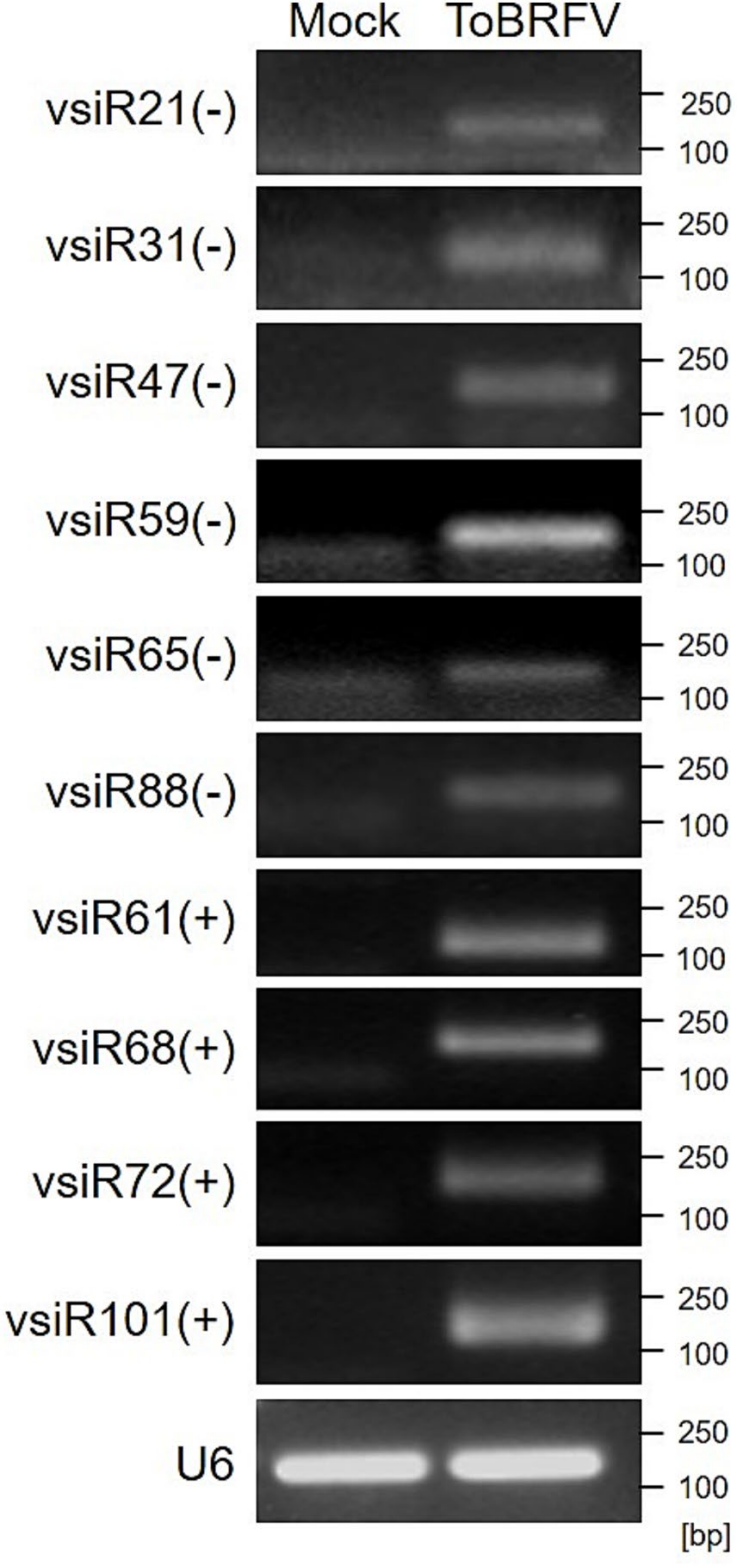
Detection of vsiRNAs in ToBRFV-infected tomato plants using RT-PCR. Different vsiRNAs were detected in tomato samples from mock-inoculated and ToBRFV-inoculated plants using RT-PCR. Tomato U6 SnRNA was used as the reference gene. “+” indicates vsiRNAs derived from the sense strand of the ToBRFV genome, while “-” indicates vsiRNAs derived from the antisense strand of the ToBRFV genome.

When viral infection triggers RNA silencing, a large amount of vsiRNAs accumulates in the host plants. To confirm the presence of vsiRNAs, we selected 10 vsiRNAs with abundant reads based on high-throughput sequencing results for RT-PCR validation. The results showed that these vsiRNAs were accumulated in tomato plants infected with ToBRFV (the RNA samples used here are different from the samples used for the aforementioned small RNA sequencing, Figure 4; Supplementary Figure S2), indicating that ToBRFV infection indeed leads to a significant accumulation of vsiRNAs in the host plants.

### 3.3 Analysis of plant transcripts targeted by vsiRNAs

In previous studies, vsiRNAs have been shown to target host mRNA at the post-transcriptional level (Shimura et al., 2011; Smith et al., 2011; Navarro et al., 2012; Adkar-Purushothama et al., 2015; Yang et al., 2020). To further understand the function of vsiRNAs, we used the psRNATarget server^4^ to explore the tomato genes that may be targeted by siRNAs derived from ToBRFV. From the large number of vsiRNAs obtained through sequencing, we selected 110 vsiRNAs with more than 2000 reads for further analysis (Supplementary Table S5). The results showed that the vast majority of vsiRNAs had multiple target genes.

**Figure 5:**
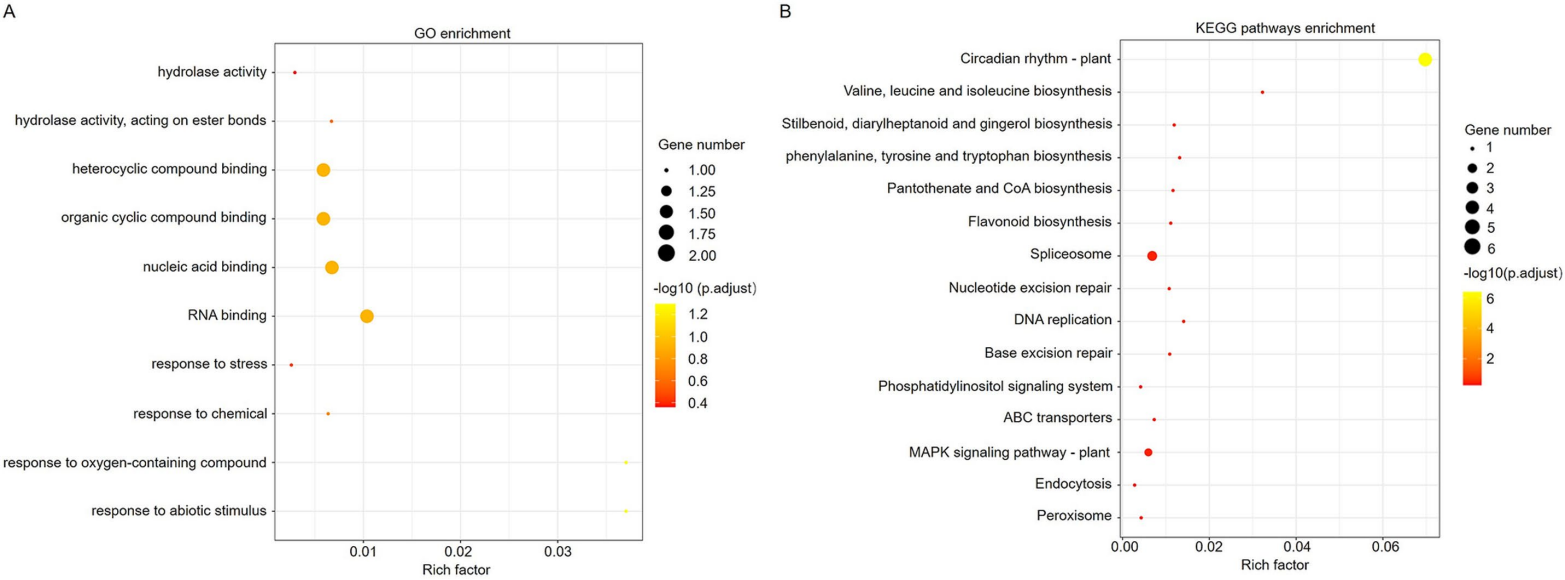
GO/KEGG analysis of the predicted vsiRNA target transcripts in tomato. **(A)** GO enrichment analysis of predicted target genes of vsiRNA in tomato. **(B)** KEGG enrichment analysis of predicted target genes of vsiRNA in tomato.

To investigate the role of ToBRFV-derived siRNA in tomato plants, we selected 248 predicted target genes with high credibility (expected value less than or equal to 2.5) for gene ontology (GO) analysis (Supplementary Table S6). The results showed that the biological process categories of the potential target genes included response to oxygen-containing compounds, response to abiotic stimulus, RNA binding, nucleic acid binding, heterocyclic compound binding, organic cyclic compound binding, and more (Figure 5A). The broad functional range of the potential target genes suggests that the identified vsiRNAs may play a very important role in the interaction between ToBRFV and tomato.

To further understand the molecular biological functions of the vsiRNA target genes, we conducted a KEGG analysis. Through pathway enrichment analysis, a total of 15 pathways were identified (Figure 5B). The potential target genes of ToBRFV-derived siRNAs mainly occur in pathways related to circadian rhythm, amino acid synthesis, flavonoid compound synthesis, spliceosome function, MAPK signaling pathway, DNA repair, and more (Figure 5B). This suggests that vsiRNAs may influence host metabolism and stress responses by regulating the expression of host genes.

**Figure 6:**
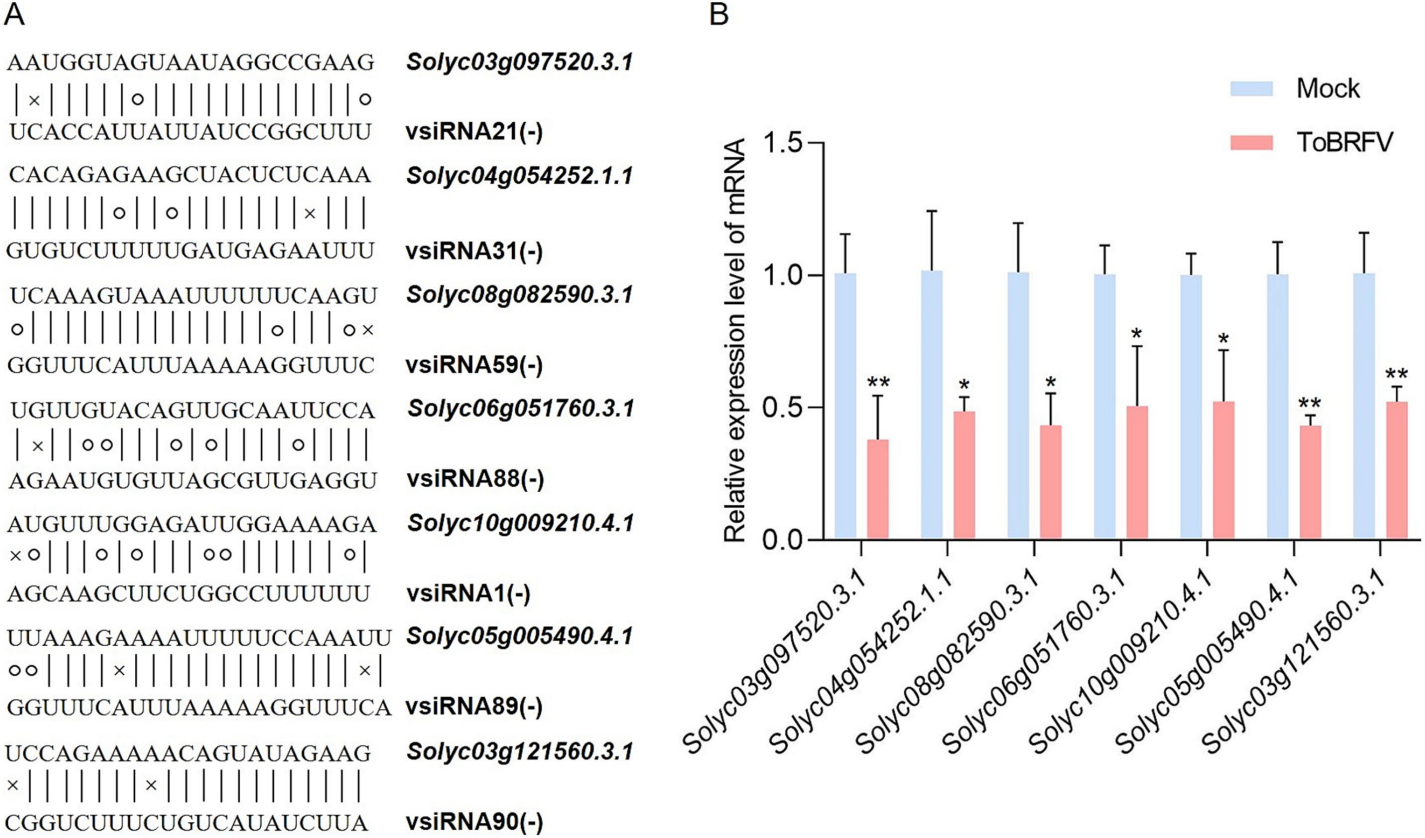
Analysis of the relative expression levels of seven putative tomato target genes after ToBRFV infection. **(A)** Sequence alignment between seven predicted target genes and vsiRNA derived from ToBRFV. Canonical base pairs are indicated by vertical lines, wobble G-U pairs are shown with circles, and non-base pairs are marked with “x.” **(B)** qRT-PCR measurement of the relative expression levels of target genes 19 days post-ToBRFV inoculation. The experimental results were similar across three repeated experiments. Data were analyzed using a Student *t*-test, with bar graphs representing mean ± SD. “*” indicates a significance level of <0.05; “**” indicates a significance level of <0.01.

In addition, we analyzed the potential target genes of vsiRNAs using quantitative fluorescence PCR. Based on the principle that vsiRNAs may downregulate the accumulation of their target genes, we screened several target genes that had at most two completely mismatched nucleotides with the vsiRNAs (Figure 6A). The selected genes include histone kinase, gene of unknown function, glutathione peroxidase, arginyl-tRNA protein transferase, calmodulin-binding protein, quinone oxidoreductase, and stress response gene (Supplementary Table S7). The experimental results showed that compared to the mock-inoculated control, the seven selected transcripts were significantly downregulated in samples infected with ToBRFV (the RNA samples used here are different from those used for small RNA sequencing, Figure 6B).

## 4 Discussion

ToBRFV was first detected in April 2015 from tomato plants in a greenhouse in Jordan that had slight wrinkling of their leaves and fruits with distinct brown mottling which significantly decreased their market value (Salem et al., 2016). RNA silencing is a small RNA-mediated gene regulatory repression mechanism in eukaryotes that plays a crucial role in plant defense against viral infections (Zhu and Guo, 2012). Viral infection triggers the production of vsiRNAs in infected plant cells, and in recent years, high-throughput sequencing technology has been widely applied to the analysis of vsiRNAs (Yan et al., 2010; Mitter et al., 2013; Xia et al., 2014; Li et al., 2016; Li et al., 2017; Qiu et al., 2017; Xu and Zhou, 2017; Zhao et al., 2018; Wu et al., 2019; Lan and Lu, 2020; Jiao et al., 2022), aiding in the understanding of vsiRNAs’ roles in plant antiviral silencing mechanisms and the virus–host interaction process. However, siRNAs derived from ToBRFV have not been analyzed before. In this study, the vsiRNA population from ToBRFV-inoculated tomato plants was analyzed using deep sequencing methods.

The mock library and the ToBRFV-infected library constructed in this study contain approximately 10 million small RNA reads each (Supplementary Table S2). However, if we exclude the number of vsiRNAs in the ToBRFV-infected library, we can observe differences in endogenous small RNA reads between the mock group and the ToBRFV-infected group. This difference may be attributed to changes in endogenous small RNAs induced by ToBRFV infection. Some studies have shown that plant virus infection affects the composition of endogenous small RNA in host plants. For example, Hu et al. found that the infection of oilseed rape mosaic tobamovirus with *Arabidopsis* causes a global size-specific enrichment of miRNAs, ta-siRNAs, and other phased siRNAs (Hu et al., 2011). Through the analysis of high-throughput data, it was found that among the 18–30 nt vsiRNAs produced in tomatoes infected with ToBRFV, 21-nt and 22-nt vsiRNAs had the highest proportions, accounting for 29.6 and 16.1%, respectively, while other lengths of vsiRNAs accounted for between 1 and 10% (Figure 1B). In virus-infected plants, DCL proteins recognize, cleave, and generate vsiRNAs, which vary in length from 21 to 24 nucleotides. If we only focus on the vsiRNAs formed by plant DCL proteins (ranging from 20 to 25 nt), filtering out some of the siRNAs, the percentages of 21-nt and 22-nt vsiRNAs will increase from 29.6 and 16.1% to approximately 40.7 and 21% (Supplementary Figure S3), respectively. The high proportion of 21-nt and 22-nt vsiRNAs is consistent with other reported cases, such as sugarcane mosaic virus (SCMV; *Potyvirus sacchari*) and southern rice black-streaked dwarf virus (SRBSDV; *Fijivirus boryzae*) (Xia et al., 2014; Xu and Zhou, 2017). However, the proportion of 21-nt and 22-nt vsiRNAs for SCMV and SRBSDV reached 40–50%, indicating that the proportions of other lengths of siRNAs derived from ToBRFV are higher than those from SCMV and SRBSDV. Notably, tobacco mosaic virus (TMV; *Tobamovirus tabaci*), which is closely related to ToBRFV, had a 21-nt vsiRNA proportion of 78.4% when infecting *Arabidopsis* (Qi et al., 2009). In this study, we observed that the total proportion of 21 and 22-nt siRNAs increased in tomato leaves infected with ToBRFV compared to the mock control group, while the total proportion of 24-nt siRNAs significantly decreased (Figure 1A). This phenomenon has also been observed in pepper mild mottle virus (PMMoV; *Tobamovirus capsici*)-infected pepper leaves, cucumber green mottle mosaic virus (CGMMV; *Tobamovirus viridimaculae*)-infected gourd leaves, papaya samples infected with papaya leaf distortion mosaic virus (PLDMV; *Potyvirus caricae*), and maize samples infected with SCMV (Xia et al., 2014; Li et al., 2016; Zhao et al., 2018; Jiao et al., 2022).

In this study, among the vsiRNA library inoculated with ToBRFV, 21-nt and 22-nt vsiRNAs showed a preference for a 5′ end of U, accounting for approximately 50%. This was followed by A (approximately 20%), C (approximately 20%), and G (approximately 5%) (Figure 2A), indicating that the vast majority of ToBRFV vsiRNAs are likely to associate with AGO1. This aligns with reports on tomato spotted wilt virus (TSWV; *Orthotospovirus tomatomaculae*)-infected tomatoes, PLDMV-infected papayas, PMMoV-infected peppers, tobacco curly shoot virus (TCSV; *Begomovirus nicotianae*)-infected tobacco, and SRBSDV-infected rice (Mitter et al., 2013; Xu and Zhou, 2017; Zhao et al., 2018; Wu et al., 2019; Jiao et al., 2022). In addition, the library inoculated with ToBRFV also accumulated a large number of vsiRNAs with 5′ terminal nucleotides as A, C or G, suggesting that other AGOs are also recruited to form different RISCs that participate in antiviral defense. In recent years, relying on the rich genetic resources of *Arabidopsis thaliana*, the main steps of the antiviral RNAi process have been clarified. Research has found that some key components of known antiviral RNAi mechanisms, such as DCLs, AGOs, and RDRs, are conserved in the tomato genome. However, their antiviral functions have not been systematically studied (Ludman et al., 2023; Zhao et al., 2023). Bai et al. identified 15 Argonaute (SlAGOs) proteins in the tomato genome, among which 7 *AGO* genes (*SlAGO1a*, *1b*, *10a*, *10b*, *2a*, *4a*, and *4b*) were found to be expressed in many tissues. Genes such as *SlAGO1a*, *1b*, *4a*, and *4b* showed varying degrees of upregulation under tomato yellow leaf curl virus (TYLCV; *Begomovirus coheni*) infection and abiotic stress, indicating that tomato AGO proteins play an important role in regulating developmental signals and responding to biotic and abiotic stresses (Bai et al., 2012). Zhao et al. used CRISPR-Cas9 technology to construct *ago2a*, *ago2b*, or *ago2ab* mutants and analyzed their phenotypes after inoculation with cucumber mosaic virus (CMV; *Cucumovirus CMV*). The results showed that AGO2a could significantly inhibit the reproduction of VSR-deficient CMV and wild-type CMV-Fny in tomato, while AGO2b could not, revealing for the first time the prominent role of AGO2a in antiviral RNAi innate immunity in tomato (Zhao et al., 2023).

SiRNAs derived from the negative strand of ToBRFV were slightly more abundant than those from the positive strand (59.13% vs. 40.87%, Figure 2B). In contrast, the vsiRNAs formed in gourd leaves infected with CGMMV primarily originated from the virus-positive strand, while the vsiRNAs in gourd fruits showed a similar ratio from both strands (Li et al., 2016). For TMV-cg infecting *Arabidopsis*, 79.9% of the vsiRNAs came from the positive RNA strand (Qi et al., 2009). In PMMoV-infected pepper leaves, 60% of the vsiRNAs originated from the virus’s positive strand, while approximately 40% came from the negative strand (Jiao et al., 2022). The observed differences among related viruses may be due to the viruses themselves or to the host plants as there are reports that the same virus can produce different vsiRNA profiles when infecting different hosts (Xu et al., 2012; Mitter et al., 2013; Yang et al., 2018).

ToBRFV vsiRNA production hotspots are located at the N-terminus of p126 and within the MP and CP genes, all derived from the negative strand of ToBRFV (Figure 3). The hotspot for 21-nt vsiRNAs overlaps with a hotspot for 22-nt vsiRNAs at the genomic location (Supplementary Table S4), indicating that different DCL enzymes, although specialized, have similar targeting affinities. Analyzing the hotspots for vsiRNA production could help design artificial siRNAs targeting the viral genome. One possible reason for hotspot formation is that certain regions of viral RNA may form partial double-stranded secondary structures, which are recognized and processed by DCL (Molnár et al., 2005). We utilized RNAfold to predict the potential secondary structures formed by the negative strand of the ToBRFV genome and found that the negative strand of the ToBRFV genome can form many partially double-stranded secondary structures (Supplementary Figures S4A,B). Four hotspots are located in the predicted imperfect duplex regions (Supplementary Figures S4C–E). In addition, it is still unclear whether the vsiRNAs formed at these hotspots have specific roles in the pathogenicity of ToBRFV or in suppressing the host’s defense responses, which requires further investigation.

Current research indicates that vsiRNAs can post-transcriptionally target host mRNAs to promote symptoms of viral infection or facilitate viral invasion (Shimura et al., 2011; Smith et al., 2011; Navarro et al., 2012; Adkar-Purushothama et al., 2015; Yang et al., 2020). Zhang et al. discovered that a siRNA derived from rice stripe virus (RSV; *Tenuivirus oryzaclavatae*) can target and cleave the *eIF4A* gene in *N. benthamiana* and rice, which negatively regulates autophagy activation through interaction with ATG5, thereby activating the antiviral autophagy pathway in both *N. benthamiana* and rice (Zhang et al., 2021). In this study, we identified a set of high-abundance vsiRNA target genes through bioinformatics analysis. RT-qPCR results showed that the transcript levels of seven potential target genes were significantly downregulated after ToBRFV infection (Figure 6B). These findings suggest that ToBRFV-derived siRNAs may promote the degradation of target transcripts, thereby creating conditions favorable for viral infection. The analysis of vsiRNAs will enhance our understanding of the interactions between ToBRFV and its host. Given the potential roles of vsiRNAs in regulating host gene expression and ToBRFV pathogenicity, the relationships between these vsiRNAs and their targets require further in-depth study.

However, our analysis also revealed that some predicted target genes did not show significant downregulation of transcript accumulation in virus-infected samples (Supplementary Figure S5), and the reasons behind this could be multifaceted. First, viral-encoded silencing suppressors may limit the regulatory potential of vsiRNAs on host targets. Second, vsiRNAs may regulate host targets not only by cleavage but also by repressing mRNA translation, similar to plant miRNAs. Finally, ToBRFV infection may induce significant increases in the transcript levels of certain target genes through other pathways.

## 5 Conclusion

In this study, we conducted the first comprehensive analysis of the small RNA profile in ToBRFV-infected tomato plants using high-throughput sequencing. We found that vsiRNAs exhibited a preference for U nucleotides at their 5′ ends, and the proportion of vsiRNAs derived from the viral genome’s negative strand was slightly higher than that from the positive strand. The vsiRNA hotspots were distributed within the RdRp N-terminus and the coding regions for MP and CP. We confirmed the presence of vsiRNAs in ToBRFV-infected tomato plants through RT-PCR. In addition, we performed GO and KEGG analyses on the host transcripts potentially targeted by the vsiRNAs, revealing that the vsiRNA target genes are involved in biological processes such as responses to biotic/abiotic stresses and binding of macromolecules such as RNA. We selected seven potential target genes for qRT-PCR analysis, confirming that their transcript accumulation levels were significantly reduced in the leaves of ToBRFV-infected tomatoes. These findings contribute to the exploration of the molecular mechanisms of vsiRNAs in the interaction between ToBRFV and tomato plants.

## Data Availability

Sequencing data from this article were deposited at the CNGB Sequence Archive (CNSA) of China National GeneBank DataBase (CNGBdb) with accession number CNP0006172.

## Footnotes

^1^ https://www.zhaolab.org/psRNATarget/

^2^ https://solgenomics.net/ftp/genomes/Solanum_lycopersicum/Heinz1706/annotation/ITAG4.0_release/

^3^ https://solgenomics.net/

^4^ https://www.zhaolab.org/psRNATarget/

## Supplementary material

SUPPLEMENTARY FIGURE S1 PCA analysis chart of 6 samples.

SUPPLEMENTARY FIGURE S2 A full scan of the entire original gels of Figure 4.

SUPPLEMENTARY FIGURE S3 Recalculation of the proportion of 21-22 nt vsiRNA.

SUPPLEMENTARY FIGURE S4 Predicting the possible RNA secondary structures formed by the negative strand of the ToBRFV genome using RNAfold. **(A)** The secondary structure predictions of the ToBRFV genome negative strand using RNAfold. **(B)** The mountain plot of the ToBRFV genome negative strand secondary structure. **(C)** Secondary structure of nucleotides 679-699 of the viral negative strand. **(D)** Secondary structure of nucleotides 837-859 of the viral negative strand. **(E)** Secondary structure of nucleotides 6160-6180 of the viral negative strand. The red boxes marked as 1, 2, and 3 in **A** represent the regions of four vsiRNA hotspots (corresponding to genomic nucleotide positions 5693-5713, 5533-5555, and 212-232, respectively), with their enlarged images corresponding to **C**, **D**, and **E** respectively.

SUPPLEMENTARY FIGURE S5 Some predicted vsiRNA target genes showed no significant downregulation after ToBRFV infection as detected by qRT-PCR.

## References

[ref1] Adkar-Purushothama C. R. Brosseau C. Giguère T. Sano T. Moffett P. Perreault J. P. (2015). Small RNA derived from the virulence modulating region of the potato spindle tuber viroid silences callose synthase genes of tomato plants. Plant Cell 27, 2178–2194. doi: 10.1105/tpc.15.00523, PMID: 26290537 PMC4568511

[ref2] Ahlquist P. (2002). RNA-dependent RNA polymerases, viruses, and RNA silencing. Science 296, 1270–1273. doi: 10.1126/science.1069132, PMID: 12016304

[ref3] Bai M. Yang G. S. Chen W. T. Mao Z. C. Kang H. X. Chen G. H.. (2012). Genome-wide identification of dicer-like, Argonaute and RNA-dependent RNA polymerase gene families and their expression analyses in response to viral infection and abiotic stresses in *Solanum lycopersicum*. Gene 501, 52–62. doi: 10.1016/j.gene.2012.02.009, PMID: 22406496

[ref4] Baulcombe D. (2004). RNA silencing in plants. Nature 431, 356–363. doi: 10.1038/nature02874, PMID: 15372043

[ref5] Baumberger N. Baulcombe D. C. (2005). Arabidopsis ARGONAUTE1 is an RNA slicer that selectively recruits microRNAs and short interfering RNAs. Proc. Natl. Acad. Sci. USA 102, 11928–11933. doi: 10.1073/pnas.0505461102, PMID: 16081530 PMC1182554

[ref6] Blevins T. Rajeswaran R. Shivaprasad P. V. Beknazariants D. Si-Ammour A. Park H. S.. (2006). Four plant dicers mediate viral small RNA biogenesis and DNA virus induced silencing. Nucleic Acids Res. 34, 6233–6246. doi: 10.1093/nar/gkl886, PMID: 17090584 PMC1669714

[ref7] Bouché N. Lauressergues D. Gasciolli V. Vaucheret H. (2006). An antagonistic function for Arabidopsis DCL2 in development and a new function for DCL4 in generating viral siRNAs. EMBO J. 25, 3347–3356. doi: 10.1038/sj.emboj.7601217, PMID: 16810317 PMC1523179

[ref8] Carbonell A. (2017). Plant ARGONAUTEs: features, functions, and unknowns. Methods Mol. Biol. 1640, 1–21. doi: 10.1007/978-1-4939-7165-7_1, PMID: 28608331

[ref9] Carbonell A. Carrington J. C. (2015). Antiviral roles of plant ARGONAUTES. Curr. Opin. Plant Biol. 27, 111–117. doi: 10.1016/j.pbi.2015.06.013, PMID: 26190744 PMC4618181

[ref10] Castel S. E. Martienssen R. A. (2013). RNA interference in the nucleus: roles for small RNAs in transcription, epigenetics and beyond. Nat. Rev. Genet. 14, 100–112. doi: 10.1038/nrg3355, PMID: 23329111 PMC4205957

[ref11] Deleris A. Gallego-Bartolome J. Bao J. Kasschau K. D. Carrington J. C. Voinnet O. (2006). Hierarchical action and inhibition of plant dicer-like proteins in antiviral defense. Science 313, 68–71. doi: 10.1126/science.1128214, PMID: 16741077

[ref12] Donaire L. Wang Y. Gonzalez-Ibeas D. Mayer K. F. Aranda M. A. Llave C. (2009). Deep-sequencing of plant viral small RNAs reveals effective and widespread targeting of viral genomes. Virology 392, 203–214. doi: 10.1016/j.virol.2009.07.005, PMID: 19665162

[ref13] Fusaro A. F. Matthew L. Smith N. A. Curtin S. J. Dedic-Hagan J. Ellacott G. A.. (2006). RNA interference-inducing hairpin RNAs in plants act through the viral defence pathway. EMBO Rep. 7, 1168–1175. doi: 10.1038/sj.embor.7400837, PMID: 17039251 PMC1679793

[ref14] Garcia-Ruiz H. Takeda A. Chapman E. J. Sullivan C. M. Fahlgren N. Brempelis K. J.. (2010). Arabidopsis RNA-dependent RNA polymerases and dicer-like proteins in antiviral defense and small interfering RNA biogenesis during turnip mosaic virus infection. Plant Cell 22, 481–496. doi: 10.1105/tpc.109.073056, PMID: 20190077 PMC2845422

[ref15] Hanssen I. M. Lapidot M. (2012). Major tomato viruses in the mediterranean basin. Adv. Virus Res. 84, 31–66. doi: 10.1016/B978-0-12-394314-9.00002-6, PMID: 22682165

[ref16] Hanssen I. M. Lapidot M. Thomma B. P. (2010). Emerging viral diseases of tomato crops. Mol. Plant-Microbe Interact. 23, 539–548. doi: 10.1094/MPMI-23-5-0539, PMID: 20367462

[ref17] Hu Q. Hollunder J. Niehl A. Kørner C. J. Gereige D. Windels D.. (2011). Specific impact of tobamovirus infection on the Arabidopsis small RNA profile. PLoS One 6:e19549. doi: 10.1371/journal.pone.0019549, PMID: 21572953 PMC3091872

[ref18] Jiao Y. Zhao X. Hao K. Gao X. Xing D. Wang Z.. (2022). Characterization of small interfering RNAs derived from pepper mild mottle virus in infected pepper plants by high-throughput sequencing. Virus Res. 307:198607. doi: 10.1016/j.virusres.2021.198607, PMID: 34688783

[ref19] Jones R. A. C. Naidu R. A. (2019). Global dimensions of plant virus diseases: current status and future perspectives. Annu. Rev. Virol. 6, 387–409. doi: 10.1146/annurev-virology-092818-015606, PMID: 31283443

[ref20] Lan H. H. Lu L. M. (2020). Characterization of hibiscus latent fort pierce virus-derived siRNAs in infected *hibiscus rosa-sinensis* in China. Plant Pathol. J. 36, 618–627. doi: 10.5423/PPJ.OA.09.2020.0169, PMID: 33312097 PMC7721542

[ref21] Li M. Li Y. Xia Z. Di D. Zhang A. Miao H.. (2017). Characterization of small interfering RNAs derived from Rice black streaked dwarf virus in infected maize plants by deep sequencing. Virus Res. 228, 66–74. doi: 10.1016/j.virusres.2016.11.001, PMID: 27888127

[ref22] Li J. Zheng H. Zhang C. Han K. Wang S. Peng J.. (2016). Different virus-derived siRNAs profiles between leaves and fruits in cucumber green mottle mosaic virus-infected *Lagenaria siceraria* plants. Front. Microbiol. 7:1797. doi: 10.3389/fmicb.2016.01797, PMID: 27881977 PMC5101232

[ref23] Livak K. J. Schmittgen T. D. (2001). Analysis of relative gene expression data using real-time quantitative PCR and the 2(-Delta Delta C(T)) method. Methods 25, 402–408. doi: 10.1006/meth.2001.1262, PMID: 11846609

[ref24] Ludman M. Szalai G. Janda T. Fátyol K. (2023). Hierarchical contribution of Argonaute proteins to antiviral protection. J. Exp. Bot. 74, 6760–6772. doi: 10.1093/jxb/erad327, PMID: 37603044 PMC10662219

[ref25] Luria N. Smith E. Reingold V. Bekelman I. Lapidot M. Levin I.. (2017). A new israeli tobamovirus isolate infects tomato plants harboring *Tm22* resistance genes. PLoS One 12:e0170429. doi: 10.1371/journal.pone.0170429, PMID: 28107419 PMC5249172

[ref26] Martínez de Alba A. E. Elvira-Matelot E. Vaucheret H. (2013). Gene silencing in plants: a diversity of pathways. Biochim. Biophys. Acta 1829, 1300–1308. doi: 10.1016/j.bbagrm.2013.10.005, PMID: 24185199

[ref27] Mi S. Cai T. Hu Y. Chen Y. Hodges E. Ni F.. (2008). Sorting of small RNAs into Arabidopsis argonaute complexes is directed by the 5′ terminal nucleotide. Cell 133, 116–127. doi: 10.1016/j.cell.2008.02.034, PMID: 18342361 PMC2981139

[ref28] Mitter N. Koundal V. Williams S. Pappu H. (2013). Differential expression of tomato spotted wilt virus-derived viral small RNAs in infected commercial and experimental host plants. PLoS One 8:e76276. doi: 10.1371/journal.pone.0076276, PMID: 24143182 PMC3797105

[ref29] Molnár A. Csorba T. Lakatos L. Várallyay E. Lacomme C. Burgyán J. (2005). Plant virus-derived small interfering RNAs originate predominantly from highly structured single-stranded viral RNAs. J. Virol. 79, 7812–7818. doi: 10.1128/JVI.79.12.7812-7818.2005, PMID: 15919934 PMC1143663

[ref30] Navarro B. Gisel A. Rodio M. E. Delgado S. Flores R. Di Serio F. (2012). Small RNAs containing the pathogenic determinant of a chloroplast-replicating viroid guide the degradation of a host mRNA as predicted by RNA silencing. Plant J. 70, 991–1003. doi: 10.1111/j.1365-313X.2012.04940.x, PMID: 22332758

[ref31] Pumplin N. Voinnet O. (2013). RNA silencing suppression by plant pathogens: defence, counter-defence and counter-counter-defence. Nat. Rev. Microbiol. 11, 745–760. doi: 10.1038/nrmicro3120, PMID: 24129510

[ref32] Qi X. Bao F. S. Xie Z. (2009). Small RNA deep sequencing reveals role for *Arabidopsis thaliana* RNA-dependent RNA polymerases in viral siRNA biogenesis. PLoS One 4:e4971. doi: 10.1371/journal.pone.0004971, PMID: 19308254 PMC2654919

[ref33] Qiu Y. Zhang Y. Hu F. Zhu S. (2017). Characterization of siRNAs derived from cucumber mosaic virus in infected tobacco plants. Arch. Virol. 162, 2077–2082. doi: 10.1007/s00705-017-3335-z, PMID: 28349357

[ref34] Quinet M. Angosto T. Yuste-Lisbona F. J. Blanchard-Gros R. Bigot S. Martinez J. P.. (2019). Tomato fruit development and metabolism. Front. Plant Sci. 10:1554. doi: 10.3389/fpls.2019.01554, PMID: 31850035 PMC6895250

[ref35] Salem N. M. Jewehan A. Aranda M. A. Fox A. (2023). Tomato brown rugose fruit virus pandemic. Annu. Rev. Phytopathol. 61, 137–164. doi: 10.1146/annurev-phyto-021622-120703, PMID: 37268006

[ref36] Salem N. Mansour A. Ciuffo M. Falk B. W. Turina M. (2016). A new tobamovirus infecting tomato crops in Jordan. Arch. Virol. 161, 503–506. doi: 10.1007/s00705-015-2677-7, PMID: 26586328

[ref37] Shabalina S. A. Koonin E. V. (2008). Origins and evolution of eukaryotic RNA interference. Trends Ecol. Evol. 23, 578–587. doi: 10.1016/j.tree.2008.06.005, PMID: 18715673 PMC2695246

[ref38] Shimura H. Pantaleo V. Ishihara T. Myojo N. Inaba J. Sueda K.. (2011). A viral satellite RNA induces yellow symptoms on tobacco by targeting a gene involved in chlorophyll biosynthesis using the RNA silencing machinery. PLoS Pathog. 7:e1002021. doi: 10.1371/journal.ppat.1002021, PMID: 21573143 PMC3088725

[ref39] Smith N. A. Eamens A. L. Wang M. B. (2011). Viral small interfering RNAs target host genes to mediate disease symptoms in plants. PLoS Pathog. 7:e1002022. doi: 10.1371/journal.ppat.1002022, PMID: 21573142 PMC3088724

[ref40] Szittya G. Moxon S. Pantaleo V. Toth G. Rusholme Pilcher R. L. Moulton V.. (2010). Structural and functional analysis of viral siRNAs. PLoS Pathog. 6:e1000838. doi: 10.1371/journal.ppat.1000838, PMID: 20368973 PMC2848561

[ref41] Wang X. B. Jovel J. Udomporn P. Wang Y. Wu Q. Li W. X.. (2011). The 21-nucleotide, but not 22-nucleotide, viral secondary small interfering RNAs direct potent antiviral defense by two cooperative argonautes in *Arabidopsis thaliana*. Plant Cell 23, 1625–1638. doi: 10.1105/tpc.110.082305, PMID: 21467580 PMC3101545

[ref42] Wang X. B. Wu Q. Ito T. Cillo F. Li W. X. Chen X.. (2009). RNAi-mediated viral immunity requires amplification of virus-derived siRNAs in *Arabidopsis thaliana*. Proc. Natl. Acad. Sci. USA 107, 484–489. doi: 10.1073/pnas.0904086107, PMID: 19966292 PMC2806737

[ref43] Weiberg A. Jin H. (2015). Small RNAs—the secret agents in the plant–pathogen interactions. Curr. Opin. Plant Biol. 26, 87–94. doi: 10.1016/j.pbi.2015.05.033, PMID: 26123395 PMC4573252

[ref44] Wu G. Hu Q. Du J. Li K. Sun M. Jing C.. (2019). Molecular characterization of virus-derived small RNAs in *Nicotiana benthamiana* plants infected with tobacco curly shoot virus and its β satellite. Virus Res. 265, 10–19. doi: 10.1016/j.virusres.2019.02.017, PMID: 30831178

[ref45] Xia Z. Peng J. Li Y. Chen L. Li S. Zhou T.. (2014). Characterization of small interfering RNAs derived from sugarcane mosaic virus in infected maize plants by deep sequencing. PLoS One 9:e97013. doi: 10.1371/journal.pone.0097013, PMID: 24819114 PMC4018358

[ref46] Xu Y. Huang L. Fu S. Wu J. Zhou X. (2012). Population diversity of rice stripe virus-derived siRNAs in three different hosts and RNAi-based antiviral immunity in *Laodelphgax striatellus*. PLoS One 7:e46238. doi: 10.1371/journal.pone.0046238, PMID: 23029445 PMC3460854

[ref47] Xu D. Zhou G. (2017). Characteristics of siRNAs derived from southern rice black-streaked dwarf virus in infected rice and their potential role in host gene regulation. Virol. J. 14:27. doi: 10.1186/s12985-017-0699-3, PMID: 28183327 PMC5301327

[ref48] Yan F. Zhang H. Adams M. J. Yang J. Peng J. Antoniw J. F.. (2010). Characterization of siRNAs derived from rice stripe virus in infected rice plants by deep sequencing. Arch. Virol. 155, 935–940. doi: 10.1007/s00705-010-0670-8, PMID: 20396917

[ref49] Yang M. Xu Z. Zhao W. Liu Q. Li Q. Lu L.. (2018). Rice stripe virus-derived siRNAs play different regulatory roles in rice and in the insect vector *Laodelphax striatellus*. BMC Plant Biol. 18:219. doi: 10.1186/s12870-018-1438-7, PMID: 30286719 PMC6172784

[ref50] Yang J. Zhang T. Li J. Wu N. Wu G. Yang J.. (2020). Chinese wheat mosaic virus-derived vsiRNA-20 can regulate virus infection in wheat through inhibition of vacuolar- (H(+))-PPase induced cell death. New Phytol. 226, 205–220. doi: 10.1111/nph.16358, PMID: 31815302 PMC7065157

[ref51] Zhang S. Griffiths J. S. Marchand G. Bernards M. A. Wang A. (2022). Tomato brown rugose fruit virus: An emerging and rapidly spreading plant RNA virus that threatens tomato production worldwide. Mol. Plant Pathol. 23, 1262–1277. doi: 10.1111/mpp.13229, PMID: 35598295 PMC9366064

[ref52] Zhang X. Yin Y. Su Y. Jia Z. Jiang L. Lu Y.. (2021). eIF4A, a target of siRNA derived from rice stripe virus, negatively regulates antiviral autophagy by interacting with ATG5 in *Nicotiana benthamiana*. PLoS Pathog. 17:e1009963. doi: 10.1371/journal.ppat.1009963, PMID: 34587220 PMC8504976

[ref53] Zhao L. Chen Y. Xiao X. Gao H. Cao J. Zhang Z.. (2023). AGO2a but not AGO2b mediates antiviral defense against infection of wild-type cucumber mosaic virus in tomato. Hortic. Res. 10:uhad043. doi: 10.1093/hr/uhad043, PMID: 37188058 PMC10177002

[ref54] Zhao G. Tuo D. Yan P. Li X. Zhou P. Shen W. (2018). Profile of siRNAs derived from green fluorescent protein (GFP)-tagged papaya leaf distortion mosaic virus in infected papaya plants. Virus Genes 54, 833–839. doi: 10.1007/s11262-018-1601-0, PMID: 30218292

[ref55] Zhu H. Guo H. (2012). The role of virus-derived small interfering RNAs in RNA silencing in plants. Sci. China Life Sci. 55, 119–125. doi: 10.1007/s11427-012-4281-3, PMID: 22415682

