# Supplementary figures and images for: Profiling of small RNAs derived from tomato brown rugose fruit virus in infected Solanum lycopersicum plants by deep sequencing
Source: Front Microbiol. 2025 Jan 30;15:1504861. doi: 10.3389/fmicb.2024.1504861 (PMC11821604; doi:10.3389/fmicb.2024.1504861)

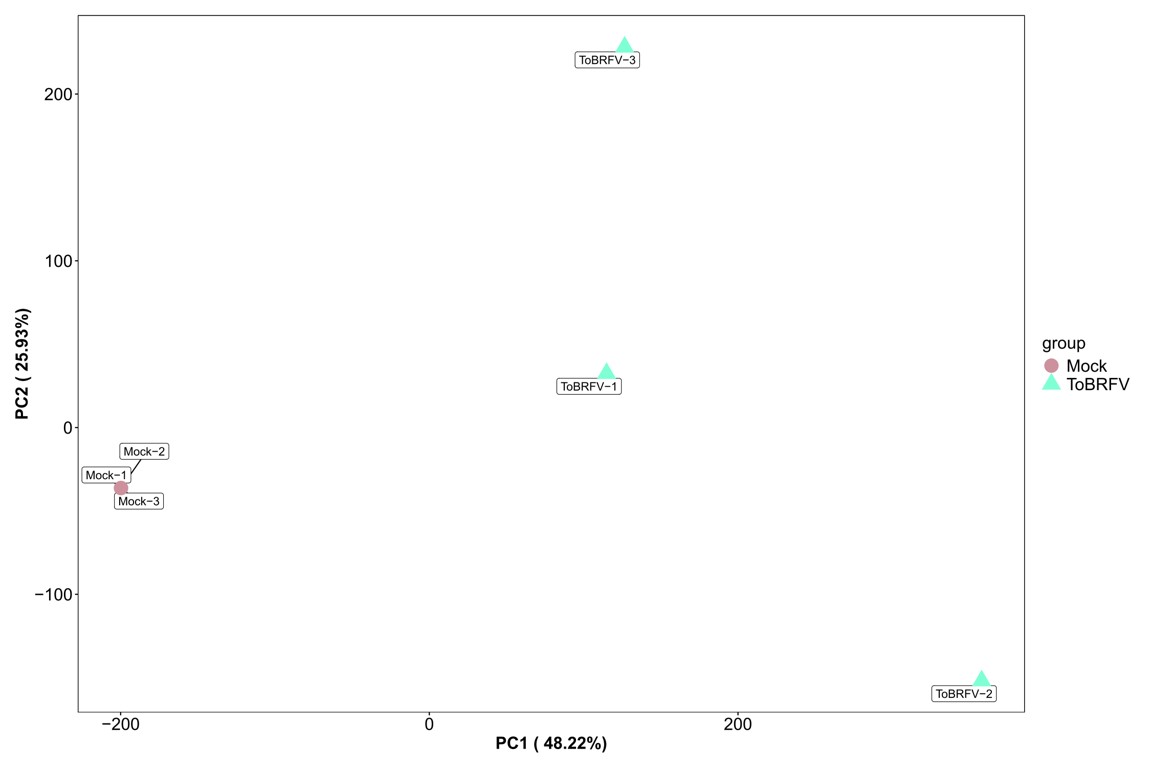

Supplement: SUPPLEMENTARY FIGURE S1 — PCA analysis chart of 6 samples. [file Image_1.jpeg]

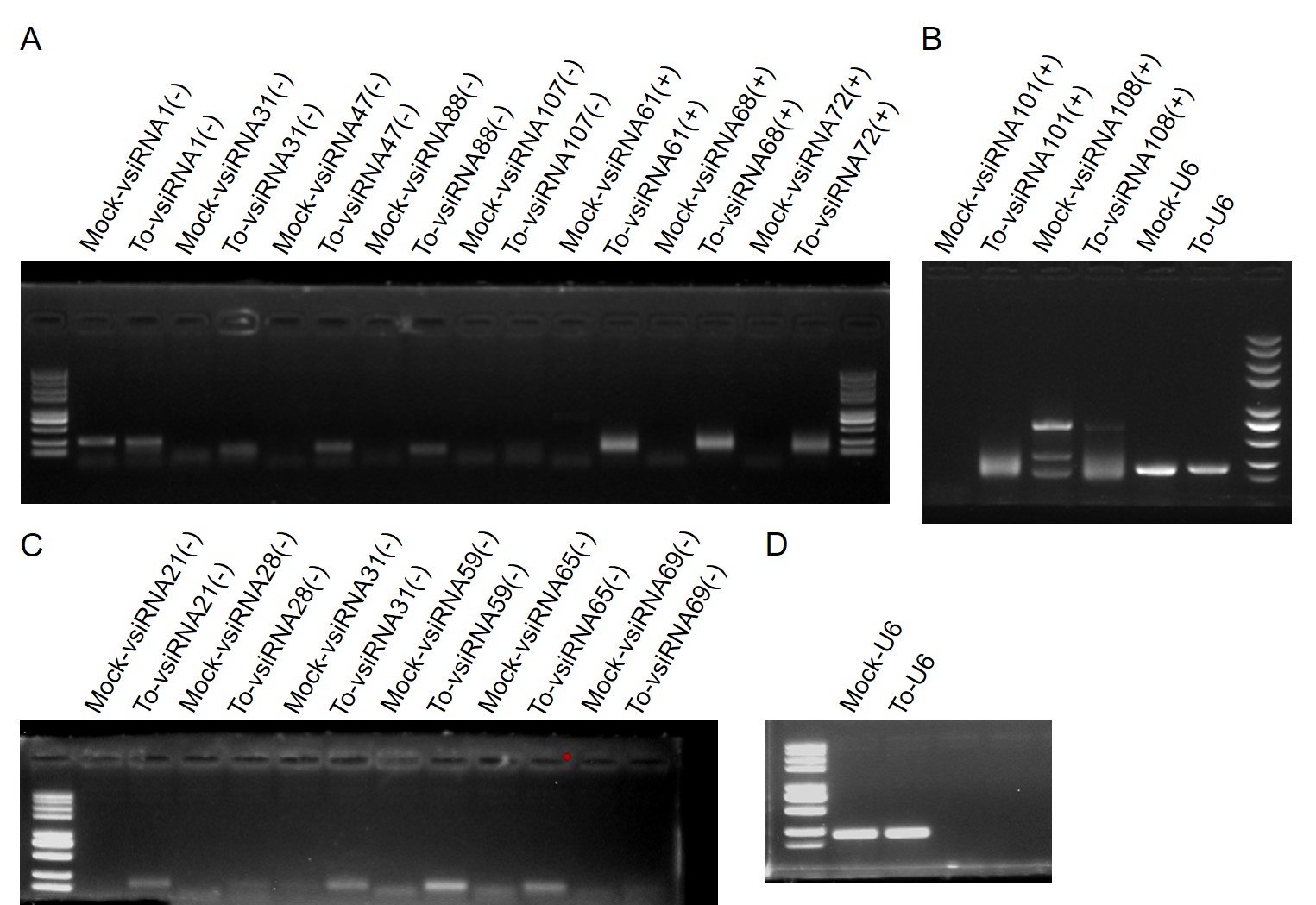

Supplement: SUPPLEMENTARY FIGURE S2 — A full scan of the entire original gels of Figure 4. [file Image_2.jpeg]

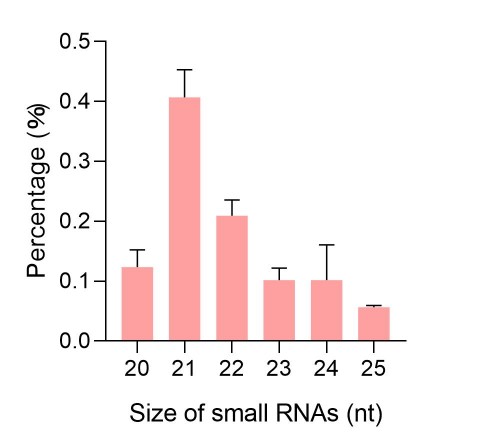

Supplement: SUPPLEMENTARY FIGURE S3 — Recalculation of the proportion of 21-22 nt vsiRNA. [file Image_3.jpeg]

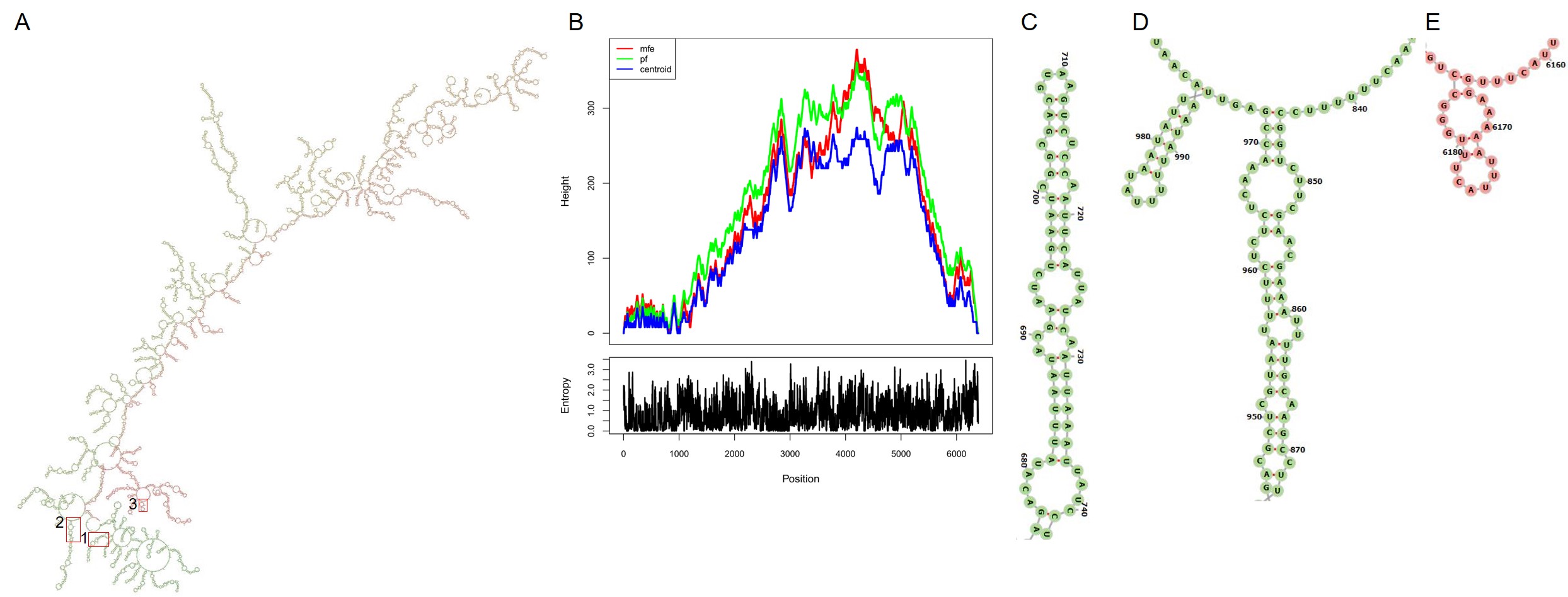

Supplement: SUPPLEMENTARY FIGURE S4 — Predicting the possible RNA secondary structures formed by the negative strand of the ToBRFV genome using RNAfold. (A) The secondary structure predictions of the ToBRFV genome negative strand using RNAfold. (B) The mountain plot of the ToBRFV genome negative strand secondary structure. (C) Secondary structure of nucleotides 679-699 of the viral negative strand. (D) Secondary structure of nucleotides 837-859 of the viral negative strand. (E) Secondary structure of nucleotides 6160-6180 of the viral negative strand. The red boxes marked as 1, 2, and 3 in A represent the regions of four vsiRNA hotspots (corresponding to genomic nucleotide positions 5693-5713, 5533-5555, and 212-232, respectively), with their enlarged images corresponding to C, D, and E respectively. [file Image_4.jpeg]

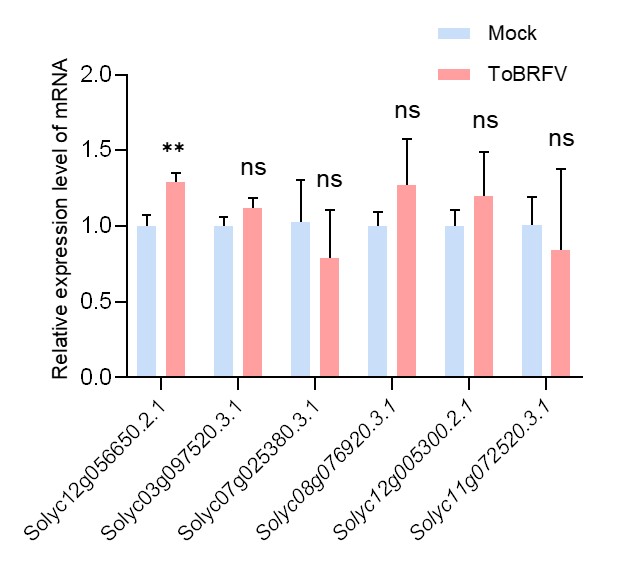

Supplement: SUPPLEMENTARY FIGURE S5 — Some predicted vsiRNA target genes showed no significant downregulation after ToBRFV infection as detected by qRT-PCR. [file Image_5.jpeg]
