# Supplementary material for: Profiling of small RNAs derived from tomato brown rugose fruit virus in infected Solanum lycopersicum plants by deep sequencing
Source: Front Microbiol. 2025 Jan 30;15:1504861. doi: 10.3389/fmicb.2024.1504861 (PMC11821604; doi:10.3389/fmicb.2024.1504861)
Supplement: Supplementary file 12 [file Table_7.docx]

Table S7. Functional annotation of 7 potential vsiRNA target genes.

|  | Gene name | Function description |
| --- | --- | --- |
|  | *Solyc03g097520.3.1* | Histidine kinase and HSP90-like ATPase family protein |
|  | *Solyc04g054252.1.1* | Unknown |
|  | *Solyc08g082590.3.1* | Glutaredoxin |
|  | *Solyc06g051760.3.1*  *Solyc10g009210.4.1*  *Solyc05g005490.4.1*  *Solyc03g121560.3.1* | Arginyl-tRNA--protein transferase  Calmodulin binding protein-like  Quinone oxidoreductase-like protein  CA-responsive protein |
